# Comparison of Apparent Diffusion Coefficient and Intravoxel Incoherent Motion for Differentiating among Glioblastoma, Metastasis, and Lymphoma Focusing on Diffusion-Related Parameter

**DOI:** 10.1371/journal.pone.0134761

**Published:** 2015-07-30

**Authors:** Woo Hyun Shim, Ho Sung Kim, Choong-Gon Choi, Sang Joon Kim

**Affiliations:** Department of Radiology and Research Institute of Radiology, Asan Medical Center, University of Ulsan College of Medicine, Seoul, Republic of Korea; NIH, UNITED STATES

## Abstract

**Background and Purpose:**

Brain tumor cellularity has been assessed by using apparent diffusion coefficient (ADC). However, the ADC value might be influenced by both perfusion and true molecular diffusion, and the perfusion effect on ADC can limit the reliability of ADC in the characterization of tumor cellularity, especially, in hypervascular brain tumors. In contrast, the IVIM technique estimates parameter values for diffusion and perfusion effects separately. The purpose of our study was to compare ADC and IVIM for differentiating among glioblastoma, metastatic tumor, and primary CNS lymphoma (PCNSL) focusing on diffusion-related parameter.

**Materials and Methods:**

We retrospectively reviewed the data of 128 patients with pathologically confirmed glioblastoma (n = 55), metastasis (n = 31), and PCNSL (n = 42) prior to any treatment. Two neuroradiologists independently calculated the maximum IVIM-f (f_max_) and minimum IVIM-D (D_min_) by using 16 different b-values with a bi-exponential fitting of diffusion signal decay, minimum ADC (ADC_min_) by using 0 and 1000 b-values with a mono-exponential fitting and maximum normalized cerebral blood volume (nCBV_max_). The differences in f_max_, D_min_, nCBV_max,_ and ADC_min_ among the three tumor pathologies were determined by one-way ANOVA with multiple comparisons. The f_max_ and D_min_ were correlated to the corresponding nCBV and ADC using partial correlation analysis, respectively.

**Results:**

Using a mono-exponential fitting of diffusion signal decay, the mean ADC_min_ was significantly lower in PCNSL than in glioblastoma and metastasis. However, using a bi-exponential fitting, the mean D_min_ did not significantly differ in the three groups. The mean f_max_ significantly increased in the glioblastomas (reader 1, 0.103; reader 2, 0.109) and the metastasis (reader 1, 0.105; reader 2, 0.107), compared to the primary CNS lymphomas (reader 1, 0.025; reader 2, 0.023) (*P* < .001 for each). The correlation between f_max_ and the corresponding nCBV was highest in glioblastoma group, and the correlation between D_min_ and the corresponding ADC was highest in primary CNS lymphomas group.

**Conclusion:**

Unlike ADC value derived from a mono-exponential fitting of diffusion signal, diffusion-related parametric value derived from a bi-exponential fitting with separation of perfusion effect doesn’t differ among glioblastoma, metastasis, and PCNSL.

## Introduction

Diffusion-weighted imaging (DWI) is a non contrast-enhanced type of magnetic resonance imaging (MRI) which is most simply performed with two b values, such as 0 and 1000 s/mm^2^. The exponential decay of signals is proposed based on the assumption of the monoexponential fit to arrive at a decay constant, referred to as the apparent diffusion coefficient (ADC) value. In DWI, signal attenuation in tissue with increasing b values reflects tissue diffusivity and reduces the effect of tissue microcapillary perfusion. The most common quantitative evaluation of DWI is by ADC. As is already well-established, ADC is sensitive to the microscopic displacement of water molecules, which is impeded by the presence of structures on the cellular scale. Therefore, a decrease in ADC may occur due to an increase in tumor cell density [1,2]. However, ADC values are influenced by both tissue diffusivity and pseudorandom motion caused by microcapillary perfusion. Accordingly, the signal attenuation on monoexponential DWI sometimes does not represent a linear relationship and it is difficult to calculate the accurate ADC value.

In contrast, the intravoxel incoherent motion (IVIM) technique estimates parameter values for those effects separately, measuring DWI over multiple b values and employing bi-exponential fitting. The interest in IVIM MRI, in which diffusion is modeled by a Gaussian function in order to obtain perfusion as well as diffusion information on lesions, has recently increased. Originally proposed by Le Bihan et al., IVIM imaging is a method used to separate the signal of DWI into perfusion and true molecular diffusion components [3]. Under the assumed isotropic and random nature of the microvascular network system resulting in the incoherent motion of water in the blood, both capillary perfusion (D*, f) and true molecular diffusion (D) can be assessed using DWI with multiple b-values. In a pioneering study [4], IVIM was used to quantify perfusion in the human brain. The IVIM MRI allows the simultaneous acquisition of diffusion and perfusion parameters which reflect tumor cellularity and vascularity respectively. Moreover, the IVIM is independent of the arterial input function for parameter quantification and does not require the need for intravenous contract agent injection for the data aquisition.

Recently published reports show that the ADC derived from a mono-exponential model of DWI can differentiate glioblastoma from primary central nervous system lymphoma (PCNSL) [2,5,6]. However, a recent IVIM study regarding differentiation between glioblastoma and atypical PCNSL, demonstrated that the increase of nCBV enhanced the difference between ADC and IVIM-D within the same lesion [7]. This result indicates that perfusion effect might result in an ADC derived from a mono-exponential fitting as an overestimation of IVIM-D derived from a bi-exponential model.

In this regard, we hypothesized that the ADC value derived from a mono-exponential fitting might be influenced by both perfusion and true molecular diffusion, and the perfusion effect on ADC can limit the reliability of ADC in the characterization of tumor cellularity, especially, in hypervascular brain tumors. Therefore, we tested whether the true IVIM diffusion parameter (D), i.e. a model that separates perfusion effects, differs in hypervascular and hypovascular tumors compared to the ADC value in the same lesions. The purpose of our study is to compare ADC and IVIM for differentiating among glioblastoma, metastatic tumor, and PCNSL focusing on diffusion-related parameter.

## Materials and Methods

### Patient Population

The Institutional Review Board of Asan Medical Center approved this retrospective study and waived the need for written informed consent from the participants. A retrospective review of our institution's data base identified 392 brain tumor patients who had undergone both IVIM MRI for a bi-exponential fitting and DWI for a mono-exponential fitting between November 2012 and December 2014. Among these patients, 52 patients without pathologic confirmationand 121 patients with pathologies other than glioblastoma or metastasis or PCNSL were excluded from the study. In the remaining 219 patients, 57 patients without dynamic susceptibility contrast (DSC) perfusion MR studies and 20 patients who were treated with steroid at the time of IVIM imaging study, were excluded. 14 patients were excluded because of poor image quality associated with hemorrhage or patient motion. Finally, 128 patients were included on the basis of the following criteria: (a) pathologically confirmed glioblastoma (n = 55), metastasis (n = 31) or PCNSL (n = 42) prior to any treatment; (b) no corticosteroid administration at the time of the IVIM MRI; and (c) no significant motion-related artifact. Patients with recurrent tumors were excluded and none of the included patients had neurological disorders other than a primary neoplasm. Histopathological confirmation was obtained in all patients with 76 patients (glioblastoma, n = 38; metastasis, n = 21; PCNSL, n = 17) confirmed by gross total or partial surgical resection and 52 patients (glioblastoma, n = 17; metastasis, n = 10; PCNSL, n = 25) confirmed by stereotactic biopsy. Any of the study patients did not participate in our previous studies focusing on IVIM in atypical PCNSL patients. Of the 128 study patients, 68 were male and 60 were female. The overall mean age was 50.7 years (range, 25–83 years).

### MRI Acquisition Protocols

MR imaging studies were performed on a 3-T unit (Achieva; Philips Medical Systems, Best, The Netherlands) using an eight-channel sensitivity encoding head coil. The order of our brain tumor imaging protocol was as follows: T2-weighted imaging, FLAIR, DWI, IVIM MR imaging, pre-contrast T1-weighted imaging, DCE perfusion MR imaging, post-contrast T1-weighted imaging, and DSC perfusion MR imaging. DWI for a mono-exponential model and IVIM for a bi-exponential model were separately acquired.

DWI was acquired in three orthogonal directions and combined into a trace image. DWI was obtained with the following parameters: repetition time (TR)/echo time (TE), 3000/56 ms; diffusion gradient encoding, b = 0, 1000 s/mm^2^; field of view (FOV), 25 cm; slice thickness/gap, 5 mm/2 mm; matrix, 256 × 256; and acquisition time, thirty-nine seconds.

For the IVIM MR imaging, sixteen different b-values were acquired (0, 10, 20, 40, 60, 80, 100, 120, 140, 160, 180, 200, 300, 500, 700, and 900 s/mm^2^) in three orthogonal directions and the corresponding traces were calculated prior to contrast injection. The imaging parameters for IVIM were as follows: TR/TE, 3000 ms/72 ms; a slice thickness, 5 mm; and a matrix number, 136 × 138. A correction of eddy-current-induced distortions was accomplished using gradient pre-emphasis. Parallel imaging was performed with an acceleration factor of 2, and the total acquisition time for IVIM was 4 min 21 seconds.

Dynamic susceptibility contrast (DSC) MR perfusion imaging was performed with a gradient-echo EPI. 0.1 mmol/kg of gadoteratemeglumine (Dotarem; Guerbet, Paris, France) was administrated at a rate of 4 mL/s. The DSC study were performed with the following parameters: TR of 1407 ms / TE of 40 ms; flip angle of 35°; matrix number, 128; 20 slices; and acquisition time, one minute 30 seconds.

### IVIM Model

In biological tissue, microscopic translational motions include microcirculation of blood in the capillary network and molecular diffusion of water. Under the condition that a capillary vessels is randomly distributed within an isotropic voxel, microcirculation of the blood in capillary network can be considered as an incoherent motion. The diffusion signal decay as a function of b-values with an IVIM uses the following Eq (1) [3]:

$$\frac{{\text{S}}_{\left(\text{b}\right)}}{{\text{S}}_{0}}=(1-f)e^{-bD}+fe^{-bD*},$$ (1)

in this equation, S is the mean diffusion signal intensity and S_0_ represents the signal intensity without diffusion. D* can be defined as the pseudo-diffusion coefficient which macroscopically describes the incoherent motion of blood within the capillary network, f indicates the fraction of perfusion-related signal decay over the total incoherent diffusion signal decay within each voxel, and D as the true molecular diffusion coefficient.

### Quantification of Imaging Parameters

The ADC map was obtained by using the software incorporated into the MR imaging unit. For the ADC calculation, a simple mono-exponential fit was applied using the b-values of 0 and 1000 s/mm^2^ on a voxel-by-voxel basis.

The IVIM signal equation was fitted on a voxel-by-voxel basis using in-house Matlab-based software. Two different approaches for the bi-exponential fittings for IVIM parameters (D, D*, and f) were implemented in our in-house software. First bi-exponential approache was a full bi-exponential fit. In the second bi-exponential approach, D was initially estimated using a reduced set of high b values (> 200 s/mm^2^) because the contribution of D* can be neglected at high b-values (b ≥ 200 s/mm^2^). Then, using the resulting D as a fixed parameter, the curve was fitted for f and D* with a nonlinear regression [8]. As previous reports have shown that the second bi-exponential approach delivered the most robust and signal-to-noise–enhanced results, therefore, we used the second approach for the bi-exponential fitting of IVIM data in all patients [4,9].

nCBV was calculated by using Brain perfusion-dedicated software (NordicICE; NordicNeuroLab, Bergen, Norway). After the correction of contrast-agent leakage, the numeric integration of the time concentration curve was applied to compute the relative CBV. A voxel-based calculation of the nCBV was performed by dividing each relative CBV value by that of contralateral, normal-appearing white matter.

### Image Analyses

A rigid co-registration between parametric maps (IVIM, ADC, and nCBV) and anatomical MR images was performed. Then, segmentation of contrast-enhancing lesions was performed on three-dimensional, contrast-enhanced, T1-weighted images using an automatic thresholding technique. Consequently, necrotic or cystic areas and cerebrospinal fluid (CSF)-filled ventricles and sulci were excluded.

Before assessing potential correlations between the pathologic findings and the imaging parameters and between the each imaging parameters, we estimated the inter-reader agreement. Two readers independently drew regions-of-interest (ROIs) on the co-registered segmented contrast-enhancing tumor area in all patients, using the hot-spot method according to the following steps: 1. five ROIs were manually constructed by two neuroradiologists for segmented, non-necrotic-enhancing tumor as a reference; 2. the size of the ROIs remained constant (radius, 2.0 mm); and 3. hotspot ROIs were obtained by visually choosing the highest (maximum f, f_max_; maximum D*, D*_max_) and the lowest (minimum D, D_min_; minimum ADC, ADC_min_) tumor parametric values.

**Fig 1:**
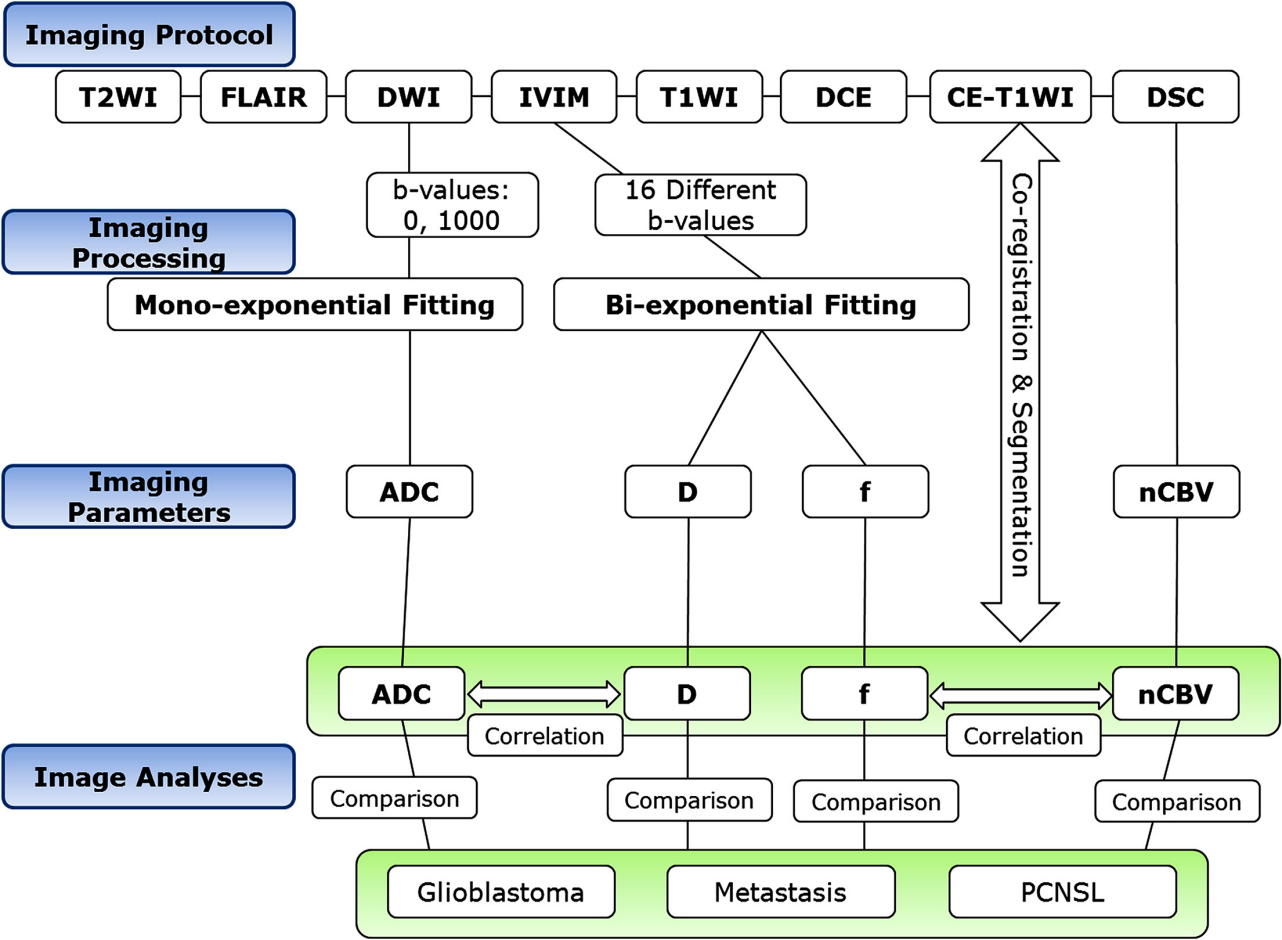
The image processing steps and workflows of imaging parameters.

For pathology and imaging correlations of IVIM parameters, analysis of the voxel-wise, calculated, parametric maps was based on hand-drawn ROIs manually placed by two neuroradiologists in consensus on the tumor area using the hot-spot method. To correlate f and D with nCBV and ADC, respectively, the nCBV and ADC values were recalculated in the corresponding ROIs of f_max_ and D_min_, respectively_._ The image processing steps and workflows of imaging parameters are shown in Fig 1.

### Statistical Analyses

All data are expressed as means ± standard deviations (SDs). Inter-reader agreement was assessed using the intraclass correlation coefficient (ICC) with 95% confidence intervals and by applying a two-way ICC with random raters assumption.

To assess the significant differences in the imaging parameters among the three pathological tumor groups, one-way ANOVA with multiple comparisons was used. The associations between f_max_ and D_min_ and the corresponding nCBV and ADC were assessed, respectively, using partial correlation analysis with adjustments made for the final pathological diagnosis. SPSS 19.0 for Windows (SPSS, Chicago, IL, USA) was used to perform all statistical analyses; *p*< 0.05 indicated statistical significance.

## Results

Image acquisition was successful in all 128 patients. The mean interval between MRI and histopathological analysis was 19.1 days. The mean time for calculation of f_max_ and D_min_ was three minutes and 57 seconds for reader 1 and three minutes and 49 seconds for reader 2.

### Visual Analysis of the IVIM MRI Parameters

**Fig 2:**
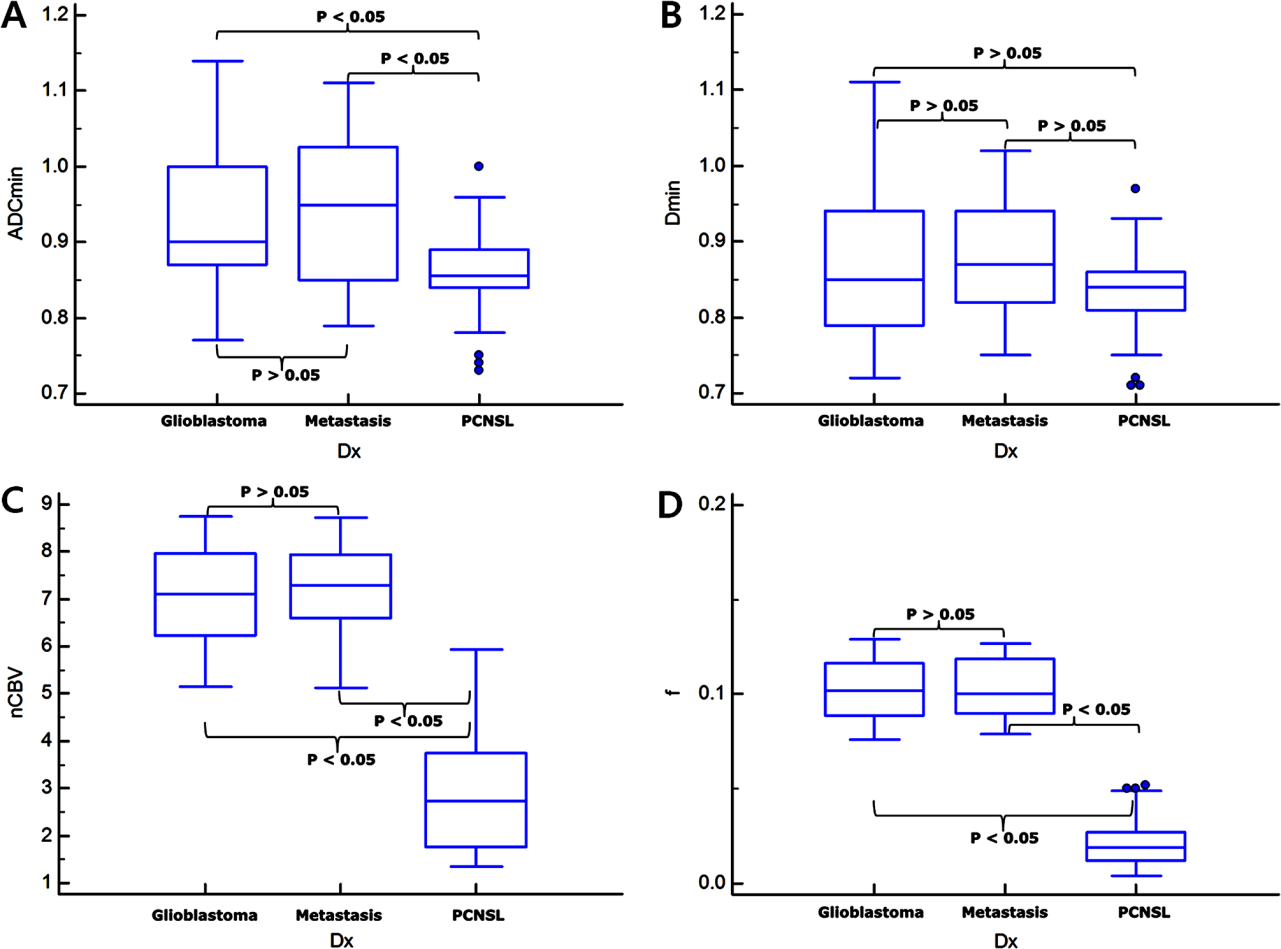
Box-and-Whisker plots for the comparisons of the imaging parameters among the three tumor groups for Reader 1. ADC_min_ (A), D_min_ (B), nCBV_max_ (C), and f_max_ (D). The P-values were calculated by using post-hoc pair-wise comparisons.

**Fig 3:**
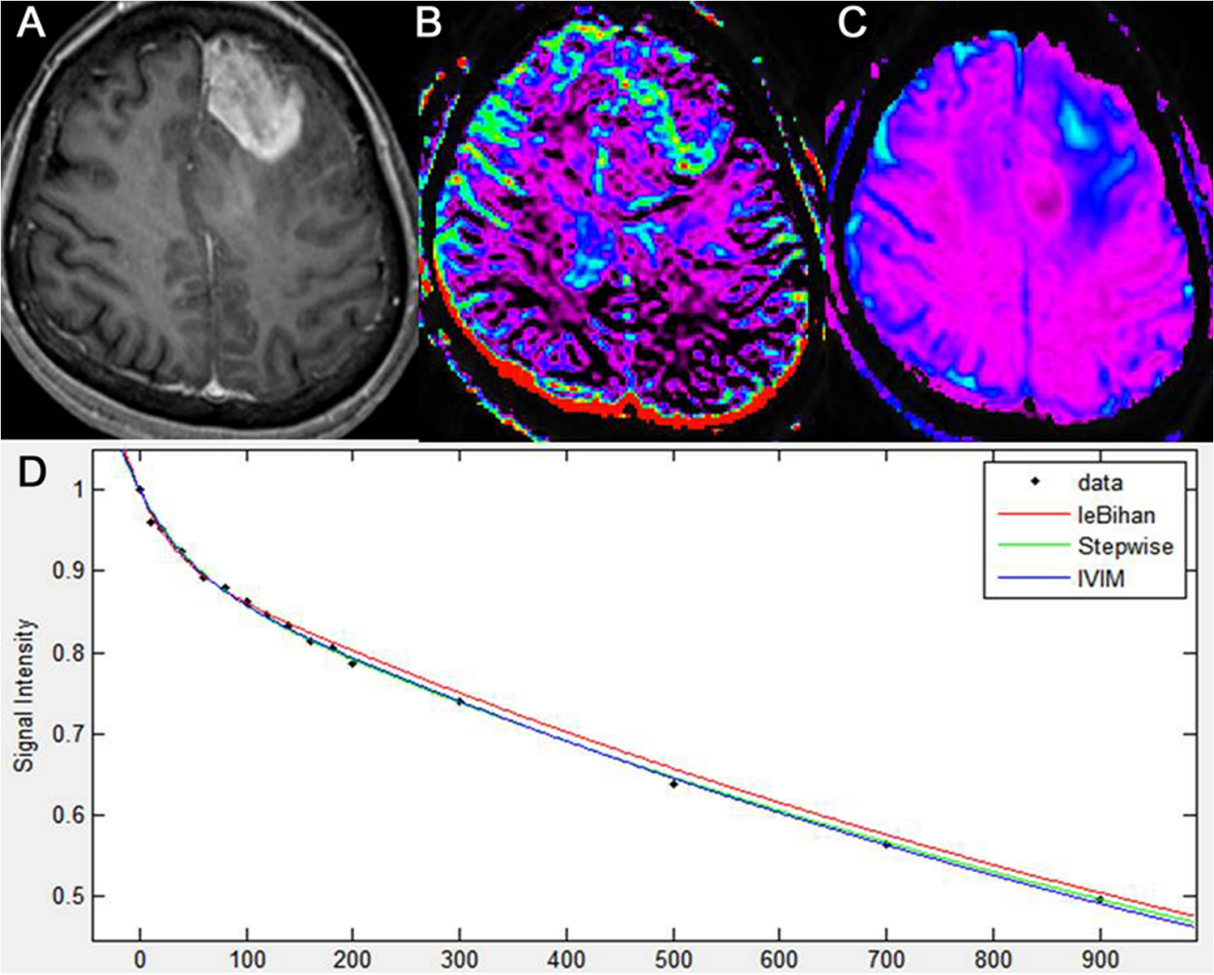
Intravoxel incoherent motion imaging of glioblastoma. Glioblastoma, centered in the left frontal lobe, as seen on axial, contrast-enhanced, T1-weighted imaging (A). IVIM-derived f shows increased perfusion in the corresponding, solid, enhancing lesion of the tumor (B). IVIM-derived D shows a similar D value to that of the surrounding, normal white matter (C). A diffusion signal decay as a function of multiple b values within the tumor solid area is biexponential (D).

All of the 55 patients with glioblastomas, all of the 31 patients with metastatic tumors, and 15 of the 42 patients with PCNSLs showed a bi-exponential pattern of diffusion signal-curve fitting in the range between 0 and 200 s/mm^2^ of the b-values. Glioblastomas and metastatic tumors showed more rapid signal decay than PCNSLs at lower b-values less than 200 s/mm^2^. The remaining 27 patients with PCNSLs demonstrated a mono-exponential pattern of diffusion signal-curve fitting in the range of 0 and 900 s/mm^2^ of b-values (Figs 1–3).

### Inter-Reader Agreement

Table 1 summarizes the inter-reader agreement using the corresponding ICCs. The inter-reader agreement was highest for nCBV_max_ (ICC, 0.91) and lowest for D*_max_ (ICC, 0.54). The ICCs between readers were higher for calculations of the perfusion parameters including f_max_ and nCBV_max_ (ICC range, 0.87–0.91) than for calculations of the diffusion parameters including D_min_ and ADC_min_ (ICC range, 0.80–0.81).

**Table 1:** Inter-reader ICC for measurement of the imaging parameters.

Parameters	Intraclass correlation coefficient^a^
f_max_	0.87 (0.79, 0.95)
D_min_	0.80 (0.65, 0.86)
D*_max_	0.54 (0.39, 0.67)
nCBV_max_	0.91 (0.81, 0.97)
ADC_min_	0.81 (0.67, 0.91)

Abbreviations: f_max_ = maximum perfusion fraction, D*_max_ = maximum pseudodiffusion coefficient, D_min_ = minimum diffusion coefficient, nCBV_max_ = maximum normalized cerebral blood volume, and ADC_min_ = minimum apparent diffusion coefficient.

^a^Numbers in parentheses are the 95% confidence intervals.

### Difference of Diffusion-Related Parameters among the Three Tumor Groups According to the Fitting Methods

The data regarding D_min_ and ADC_min_ in the three types of tumor are summarized in Table 2, and representative cases for glioblastoma, metastatic tumor, and PCNSL are shown in Figs 1–3, respectively. Using a mono-exponential fitting of diffusion signal decay, the mean ADC_min_ was significantly lower in the PCNSL group (reader 1, 0.85 ± 0.07; reader 2, 0.84 ± 0.11) than in the glioblastoma group (reader 1, 0.92 ± 0.14; reader 2, 0.93 ± 0.17) and in the metastasis group (reader 1, 0.94 ± 0.15; reader 2, 0.92 ± 0.19) (*P* = .0031–.0045). However, using a bi-exponential fitting, the mean D_min_ did not significantly differ among the three tumor groups.

**Table 2:** Subgroup analysis for correlation of the imaging parameters.

Correlation	Glioblastoma	Metastasis	Primary CNS Lymphoma	Total
f versus nCBV	0.79^a^	0.72^a^	0.57^a^	0.85^a^
D* versus nCBV	0.37	0.41	0.32	0.39
D versus ADC	0.94^a^	0.95^a^	0.98^a^	0.97^a^

Abbreviations: nCBV = normalized cerebral blood volume and ADC = apparent diffusion coefficient.

^a^ indicates statistical significance.

### Difference of Perfusion-Related Parameters among the Three Tumor Groups

**Fig 4:**
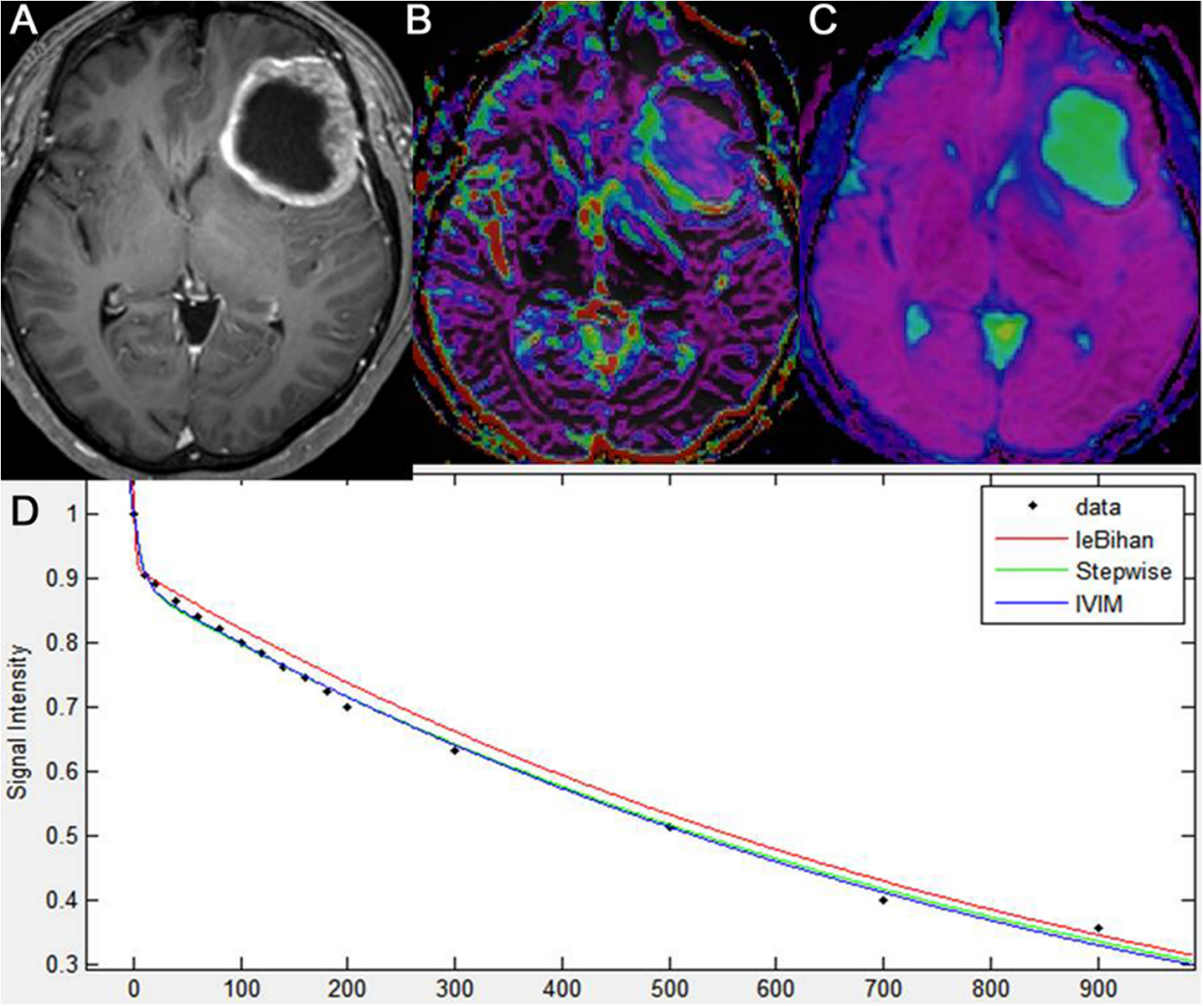
Intravoxel incoherent motion imaging of metastatic tumor. Metastatic tumor of the left frontal lobe in a patient with lung cancer, as seen on axial, contrast-enhanced T1-weighted imaging (A). IVIM-derived f shows increased perfusion in the corresponding, solid, enhancing lesion of the tumor (B). IVIM-derived D shows a similar D value to that of the surrounding, normal white matter (C). A diffusion signal decay as a function of multiple b values within the ROI of the tumor solid area is biexponential (D).

**Fig 5:**
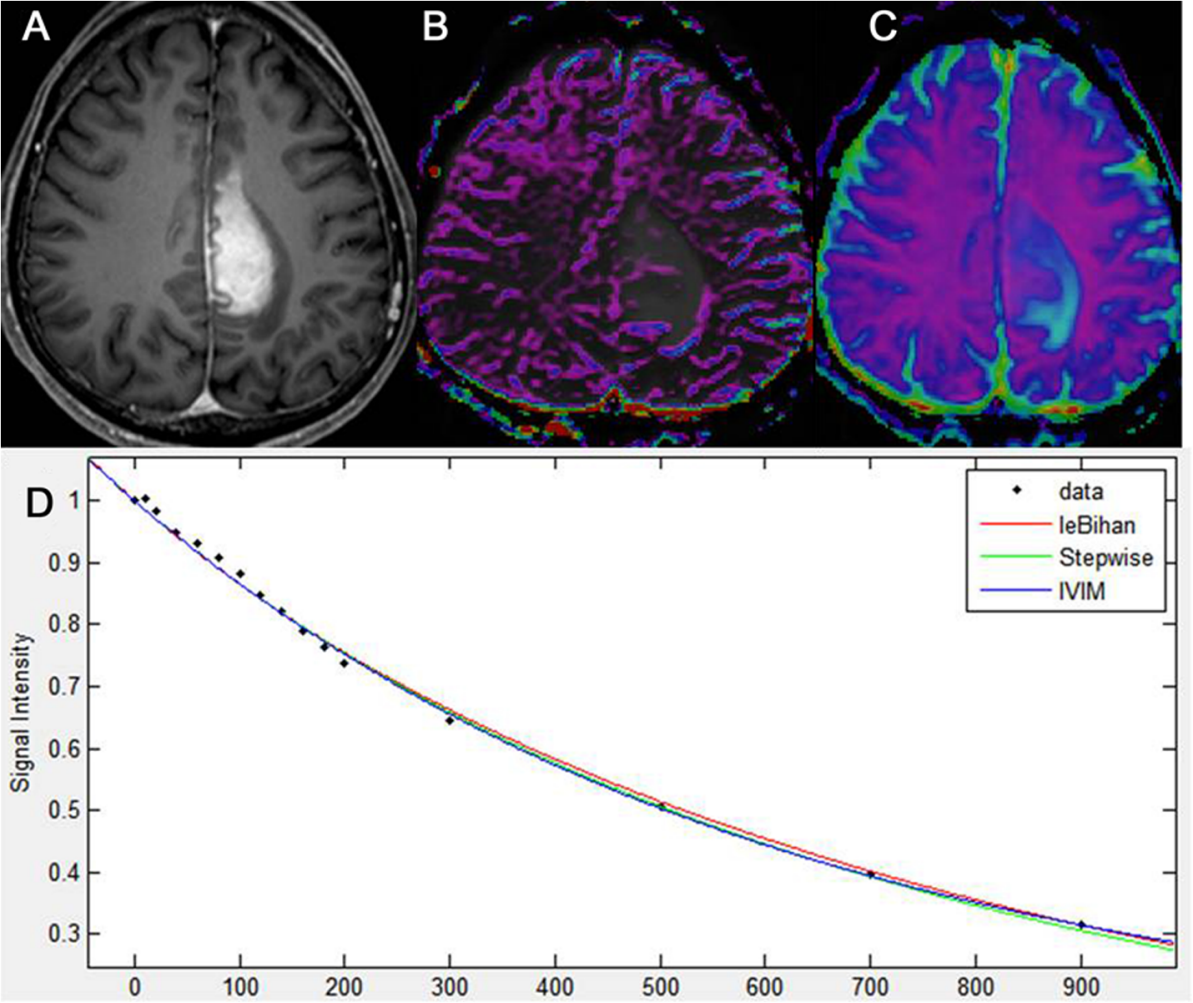
Intravoxel incoherent motion imaging of Primary CNS lymphoma. Primary CNS lymphoma of the left medial fronto-parietal lobe, as seen on axial, contrast-enhanced, T1-weighted imaging (A). IVIM-derived f shows no increase of perfusion in the corresponding, solid, enhancing lesion of the tumor (B). IVIM-derived D shows a similar D value to that of the surrounding, normal white matter (C). A diffusion signal decay as a function of multiple b values within the ROI of the tumor solid area is monoexponential (D).

The data regarding f_max_, D*_max_, and nCBV_max_ in the three types of tumor are summarized in Fig 2, and representative cases for glioblastoma, metastatic tumor, and PCNSL are shown in Figs 3–5, respectively. The mean f_max_ was significantly higher in the glioblastoma group (reader 1, 0.103 ± 0.017; reader 2, 0.109 ± 0.024) and in the metastasis group (reader 1, 0.105 ± 0.022; reader 2, 0.107 ± 0.026) than in the PCNSL group (reader 1, 0.025 ± 0.014; reader 2, 0.023 ± 0.012) (*P* < .001 for each), respectively. The mean D*_max_ was also significantly higher in the glioblastoma group (reader 1, 51.4 ± 22.9; reader 2, 57.5 ± 25.6) and in the metastasis group (reader 1, 61.1 ± 29.7; reader 2, 62.5 ± 31.2) than in the PCNSL group (reader 1, 8.2 ± 4.1; reader 2, 7.8 ± 3.9) (*P* < .001 for each).

### Correlation of the Imaging Parameters

Table 2 summarizes the correlation coefficients between f_max_ and corresponding nCBV and between D_min_ derived from bi-exponential fitting and corresponding ADC derived from mono-exponential fitting, respectively. For the tumor pathology as a controlling variable, the f_max_ value showed a significant correlation with the corresponding nCBV (r = 0.85, *P* < .001) (S1 Fig). There was also a significant correlation between D_min_ and corresponding ADC (r = 0.97; *P* < .001) (S2 Fig). However, the correlation between D*_max_ and corresponding nCBV was not significant (r = 0.39, *P* = .097). In the subgroup analyses, the correlation between f_max_ and the corresponding nCBV was highest in the glioblastoma group (r = 0.79, *P* < .001) and lowest in the PCNSL group (r = 0.57, *P* < .001). The correlation between D_min_ and the corresponding ADC was highest in the PCNSL group (r = 0.98, *P* < .001) and lowest in the glioblastoma group (r = 0.94, *P* < .001).

## Discussion

In our study, we attempted to validate the perfusion effect on the calculation of diffusion-related parameters in brain tumors by using the IVIM MRI which was derived by a bi-exponential fitting with multiple b-values. Using a mono-exponential fitting which does not consider the contribution of perfusion effect on diffusion signal decay, diffusion-related parameter (ADC_min_) was significantly lower in the PCNSL group than the other tumor groups. However, using a bi-exponential fitting which considers the contribution of perfusion effect, the mean D_min_ was not significantly different between the PCNSL and the other tumor groups. Our study also revealed that PCNSL showed significantly lower perfusion values including mean f_max_ and mean nCBV_max_ than the other tumor groups in solid-enhancing areas. These results indicate that the ADC difference between PCNSL and the other tumor groups could be associated with the contribution of perfusion effect on diffusion signal decay. In addition, D_min_ correlated best with ADC in the PCNSL group. Based on these results, we could suggest that diffusion-related parameter containing the perfusion effect may restrict the reliability of the correlation between this parameter with tumor cellularity.

Several previous studies reported that the diffusion space of water molecules in malignant tumors is limited by histopathological characteristics. This is mainly owed to hypercellularity, enlarged nuclei, and hyperchromatism, resulting in differences of ADC values between different brain tumors [2,5,6]. In previous studies, the ADC value measured on DWI using the mono-exponential model had clinical value for differentiating glioblastoma from PCNSL [2,5,6]. Guo et al. [2] reported that the mean ADC values relative to the normal white matter were significantly lower in PCNSL than in glioblastoma, thus indicating that high cellularity in PCNSL contributes to the restricted diffusion. Yamasaki et al.[5] also reported that the ADC values were significantly lower in PCNSL than in glioblastoma. Our ADC results agree with these studies.

However, high perfusion fraction in malignant brain tumors could influence the DWI signal decay in opposite directions, so IVIM-D value may be more useful in the characterization of tumor cellularity. Tissue microcirculation and cellularity contributions will influence ADC measurement in diametrically opposite directions. Sigmund et al. also concluded that tissue diffusivity, by avoiding vascular contributions and marking cellularity more precisely, provided better differentiation of normal from malignant lesions than ADC [10]. Another recent study [7] showed that D_min_ derived from IVIM was not significantly different between glioblastoma and atypical PCNSL groups, although ADC_min_ derived from a mono-exponential model, was significantly different. Moreover, there was a significant correlation between nCBV and the difference of ADC and D within the same lesion. This result might reflect that the perfusion effects could result in ADC as an overestimation of D. We validated this hypothesis by observing that D_min_ did not differ significantly in the three tumor groups and there were more significant correlations between D and ADC in the PCNSL than in the other tumor groups known as hypervascular tumors.

Accordingly, we can speculate that any ADC estimation with only two b-values, e.g. 0 and 1000 s/mm^2^, as usually performed in clinical studies, still has the perfusion effect and would miss the curvature due to perfusion contamination caused by microcirculation of blood within randomly distributed capillaries. Therefore, ADC derived from a mono-exponential fitting could be an overestimation of D derived from a bi-exponential fitting, especially in hypervascular tumors [11]. It will thus be needed when interpretating diffusion characteristics in hypervascular tumors, it should be considered that perfusion effect can contribute to the overestimation of ADC derived from the mono-exponential fitting. Further studies are needed to understand the exact pathophysiologic mechanism of our speculations.

In our study, the significant difference in f_max_ between the glioblastoma and the PCNSL groups was consistent with the results of previous studies showing that PCNSL had a lower mean relative CBV [12,13], nCBV [7] or lower rCBV_max_ values [14] than glioblastoma. Metastatic tumors usually spread into the brain via hematogenous routes and hence induce neovascularization as they grow and expand. An increase in the microvascularity and neovascularity of these tumors leads to increased rCBV [15]. In contrast to glioblastoma or metastasis, tumor neovascularization is poor in PCNSL which is well-known for its angiocentric growth pattern in which PCNSL cells tend to cluster around pre-existing brain vessels [16]; this can explain the lower f_max_ in the PCNSL patients in our study.

Application of a combination model of IVIM parameters to tissue characterization is reasonable because multiple parameters are generated in the IVIM technique. IVIM can simultaneously assess tumor vascularity and cellularity. The ability to explore both these metrics in our previous study has shown higher accuracy when compared with ADC or nCBV in discriminating tumor progression from treatment-related change [17,18]. Moreover, the inter-reader agreement was almost perfect for the f and D values, and which justifies their use in future studies and strengthens the advantage of the IVIM method as an objective and reliable parameter. Whereas, the D* values were poorer presumably due to their high sensitivity to capillary blood flow and any partial volume effect with CSF-filled or necrotic spaces. Moreover, as D* is less reproducible than f in the liver [19]. It remains a potential challenge for future studies.

Our study has several limitations. First, as the set of b values we used was not optimized, the inadequate number of the applied b values may have negatively impacted the acquired IVIM maps. However, as shown in a previously published report [20], the acquisition of 30 or more b values is not feasible due to scan time restrictions. Lemke et al.[20] suggested that at least 10 of the optimally distributed b values reported in their work should be measured in a high-quality IVIM experiment. In addition, to gain a shorter examination time without losing precise technical execution, the further optimization of b values is required. Secondly, our signal-to-noise ratio may have been below the critical value, and systematic errors, such as patient movement or partial volume effects, may also have affected the quality of the IVIM-derived parametric maps. Further improvement of both hardware and image processing will be needed in order to be able to clinically apply this method.

## Conclusions

Unlike ADC value derived from a mono-exponential fitting of diffusion signal, using a bi-exponential fitting which separate a perfusion effect from a diffusion signal, a diffusion-related parameter does not differ among glioblastoma, metastasis, and PCNSL. Therefore, the IVIM model can potentially provide more accurate information for tumor diffusion characteristics, especially in hypervascular brain tumors.

## Supporting Information

S1 Fig Scatter plots of the correlation between IVIM-derived f and the corresponding normalized CBV. There are significant correlations between f and the corresponding, normalized CBV in the glioblastoma (blue mark), metastasis (black mark), and primary CNS lymphoma (red mark) patients with tumor pathology as the controlling variable.
(TIF)

S2 Fig Scatter plots of the correlation between IVIM-derived D and the corresponding ADC. There are significant correlations between D and the corresponding ADC in the glioblastoma (blue mark), metastasis (black mark), and primary CNS lymphoma (red mark) patients using each tumor pathology factor as the controlling variable.
(TIF)
