# Supplementary figures and images for: Comparison of Apparent Diffusion Coefficient and Intravoxel Incoherent Motion for Differentiating among Glioblastoma, Metastasis, and Lymphoma Focusing on Diffusion-Related Parameter
Source: PLoS One. 2015 Jul 30;10(7):e0134761. doi: 10.1371/journal.pone.0134761 (PMC4520473; doi:10.1371/journal.pone.0134761)

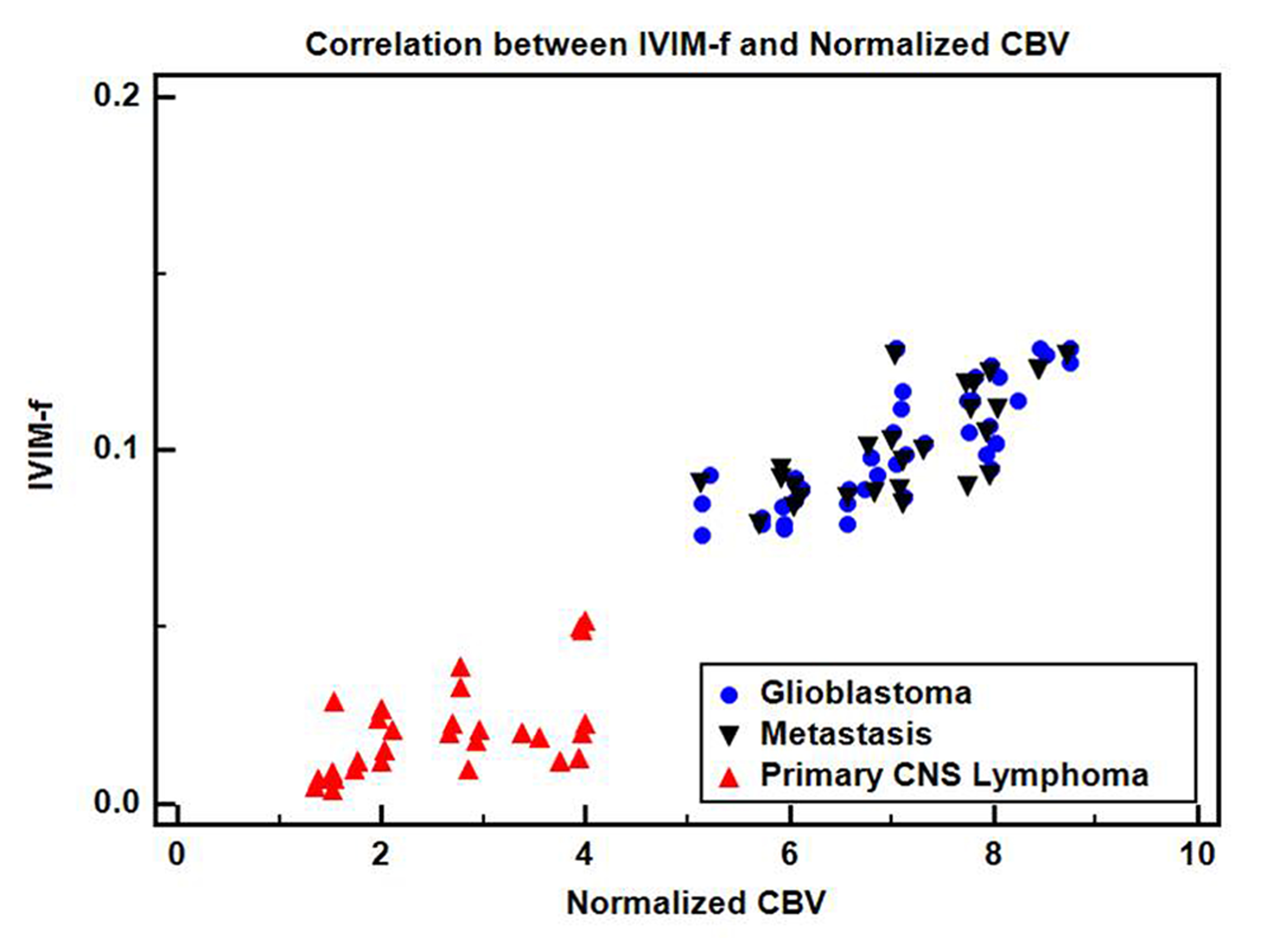

Supplement: S1 Fig — There are significant correlations between f and the corresponding, normalized CBV in the glioblastoma (blue mark), metastasis (black mark), and primary CNS lymphoma (red mark) patients with tumor pathology as the controlling variable. (TIF) [file pone.0134761.s001.tif]

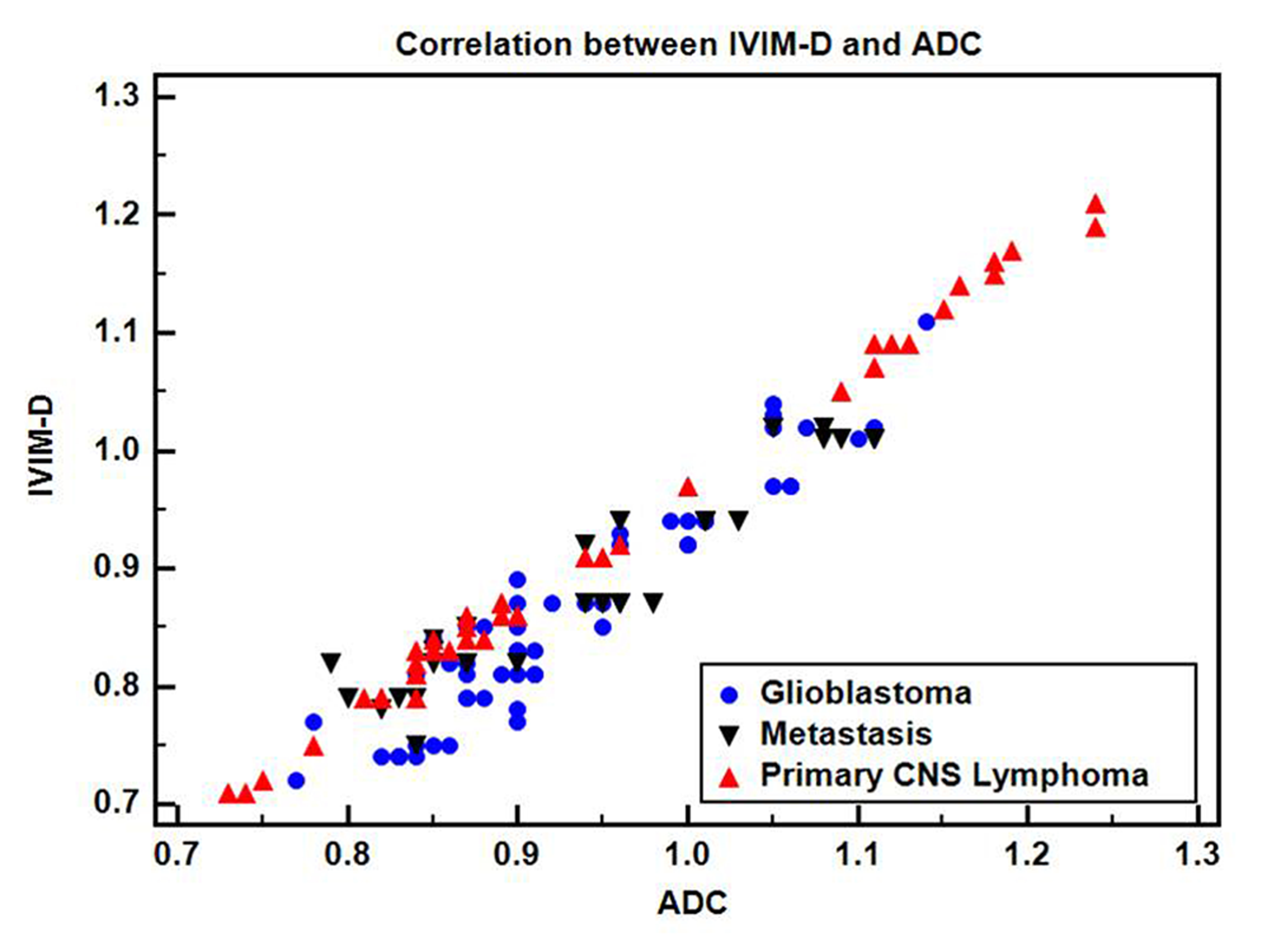

Supplement: S2 Fig — There are significant correlations between D and the corresponding ADC in the glioblastoma (blue mark), metastasis (black mark), and primary CNS lymphoma (red mark) patients using each tumor pathology factor as the controlling variable. (TIF) [file pone.0134761.s002.tif]
